# Hybrid Field-Effect Transistors and Photodetectors Based on Organic Semiconductor and CsPbI_3_ Perovskite Nanorods Bilayer Structure

**DOI:** 10.1007/s40820-018-0210-8

**Published:** 2018-06-23

**Authors:** Yantao Chen, Xiaohan Wu, Yingli Chu, Jiachen Zhou, Bilei Zhou, Jia Huang

**Affiliations:** 1 Key Laboratory of Road and Traffic Engineering of Ministry of Education, Tongji University, Shanghai, 201804 People’s Republic of China; 2 Interdisciplinary Materials Research Center, School of Materials Science and Engineering, Tongji University, Shanghai, 201804 People’s Republic of China

**Keywords:** Perovskite, Phototransistor, Nanorod, Organic semiconductor, Photogating effect

## Abstract

The outstanding performances of nanostructured all-inorganic CsPbX_3_ (X = I, Br, Cl) perovskites in optoelectronic applications can be attributed to their unique combination of a suitable bandgap, high absorption coefficient, and long carrier lifetime, which are desirable for photodetectors. However, the photosensing performances of the CsPbI_3_ nanomaterials are limited by their low charge-transport efficiency. In this study, a phototransistor with a bilayer structure of an organic semiconductor layer of 2,7-dioctyl [1] benzothieno[3,2-b] [1] benzothiophene and CsPbI_3_ nanorod layer was fabricated. The high-quality CsPbI_3_ nanorod layer obtained using a simple dip-coating method provided decent transistor performance of the hybrid transistor device. The perovskite layer efficiently absorbs light, while the organic semiconductor layer acts as a transport channel for injected photogenerated carriers and provides gate modulation. The hybrid phototransistor exhibits high performance owing to the synergistic function of the photogating effect and field effect in the transistor, with a photoresponsivity as high as 4300 A W^−1^, ultra-high photosensitivity of 2.2 × 10^6^, and excellent stability over 1 month. This study provides a strategy to combine the advantages of perovskite nanorods and organic semiconductors in fabrication of high-performance photodetectors.

**Electronic supplementary material:**

The online version of this article (10.1007/s40820-018-0210-8) contains supplementary material, which is available to authorized users.

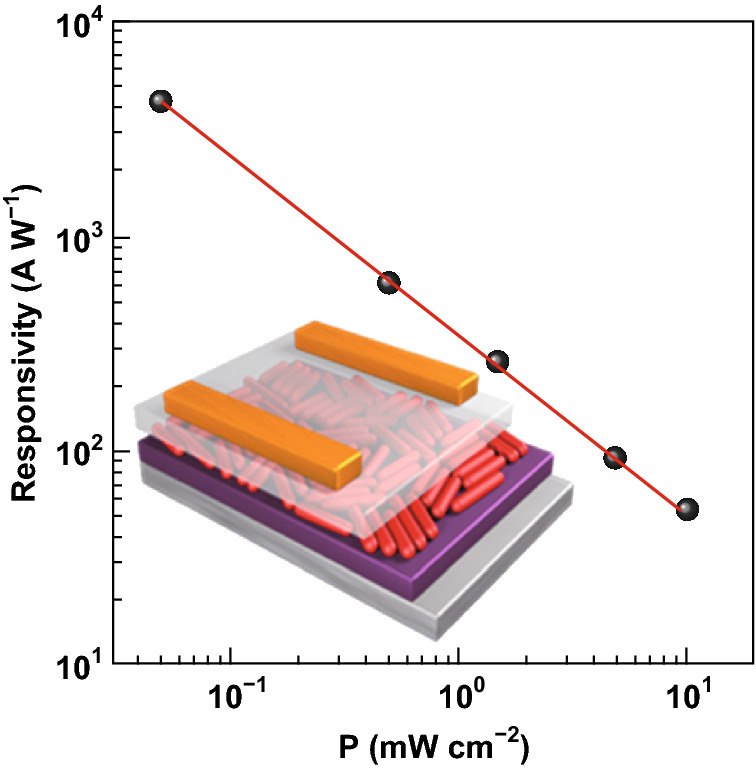

## Highlights

- A high-performance phototransistor with an organic semiconductor and CsPbI_3_ perovskite nanorod hybrid structure was fabricated and characterized.
- The perovskite layer efficiently absorbs the input illumination, while the organic semiconductor layer acts as a transport channel for injected photogenerated carriers and provides gate tuning.
- The hybrid phototransistor exhibits high performance with a photoresponsivity as high as 4300 A W^−1^, ultra-high photosensitivity of 2.2 × 10^6^, and long-term stability of 1 month.

## Introduction

Recently, organometal halide perovskites have attracted a significant interest in the field of optoelectronics owing to their desirable electronic and optoelectronic properties, such as strong and broad light absorption, weakly bound excitons, long-range-balanced electron/hole transport lengths, and low-cost fabrication [1, 2]. These characteristics make them widely use in solar cells [3, 4], light-emitting diodes (LEDs) [5, 6], lasing [7], and photodetectors [8–10]. However, these perovskite materials usually have a low long-term stability owing to the environmental degradation of organic cations within the materials from moisture and heat [11, 12]. In contrast, all-inorganic CsPbX_3_ (X = I, Br, Cl) perovskites exhibit decent stabilities; among them, the CsPbI_3_ perovskite exhibited unique properties with a suitable bandgap, high quantum efficiency, and long radiative lifetime [13, 14]. However, this material has two phases: cubic phase and orthorhombic phase. The black cubic phase is stable above 300 °C; it quickly undergoes phase transformation into the wider-bandgap (3.01 eV) orthorhombic phase upon cooling to room temperature. This phase transformation largely limited its applications. In order to overcome this problem, low-dimensional CsPbI_3_ perovskites, including quantum dots, nanowires, nanorods, and nanoplates, have been prepared. Owing to both surface and nanoeffects, as the size of the perovskite material is reduced to the nanoscale, the phase stability can significantly differ from that in the bulk counterpart [15–18].

Owing to the outstanding characteristics including few grain boundaries, large specific surface area, low charge carrier rate, long charge carrier lifetime, and high surface-to-volume ratio, one-dimensional (1D) CsPbI_3_ nanorods are promising materials for photodetector devices [19–23]. For example, Yang et al. [22] reported superior photodetectors based on a single CsPbI_3_ nanorod with an ultra-fast response and high stability. Tang et al. [23] demonstrated a simple method for the synthesis of CsPb(Br/I)_3_ nanorods; the photosensitivity of the nanorod-based photodetectors could reach up to 10^3^. Owing to the charge-transport limitation in the perovskite layers, the CsPbI_3_ nanorod-based photodetectors exhibited a relatively low photoresponsivity (*R*) [24].

Organic semiconductors (OSCs) are promising materials with large potentials in next-generation flexible electronic and photoelectronic devices owing to their attractive advantages of easily tunable bandgap, simple synthesis, low cost, and resolvability [25–31]. 2,7-Dioctyl [1] benzothieno[3,2-b] [1] benzothiophene (C8BTBT) is an excellent OSC material, which does not respond to visible light, and has a good air stability and high hole-carrier mobility [32–34]. In this study, C8BTBT/CsPbI_3_ nanorod bilayer films were produced and used to fabricate high-performance phototransistors. The gate-tunable hybrid transistor devices illuminated by white light exhibited an outstanding photosensing performance with an *R* as high as 4.3 × 10^3^ A W^−1^ and ultra-high *I*_photo_*/I*_dark_ ratio of 2.2 × 10^6^. Furthermore, the hybrid phototransistors possessed an excellent long-term stability when stored under ambient conditions for more than 1 month. Therefore, this study reveals a simple approach to fabricate hybrid phototransistors with comprehensive advantages including a simple fabrication, enhanced photosensitivity, and high stability.

## Experimental Methods

### Chemicals

C8BTBT (98%, Suna Tech Inc), Cs_2_CO_3_ (99.9%, Adamas), lead iodide (PbI_2_, 99.99%, Sigma-Aldrich), 1-octadecene (ODE, 90%, Energy Chemical), oleylamine (OA, 90%, Energy Chemical), oleylamine (OAm, 70%, Energy Chemical), and octane (99.9%, Sigma-Aldrich) were used without further purification.

### Preparation of CsPbI_3_ Nanorod

CsPbI_3_ nanorods were synthesized using the ligand-assisted method [23, 35]. Cs_2_CO_3_ (0.40 g, 1.23 mmol) was added to a 50-mL round-bottom flask along with OA (1.5 mL) and ODE (25 mL) and stirred under vacuum for 1 h at 120 °C. The flask was filled with Ar. Subsequently, all Cs_2_CO_3_ reacted with the OA when the solution was clear. PbI_2_ (1.00 g, 2.17 mmol) and ODE (50 mL) were loaded into a 150-mL round-bottom flask and dried under vacuum at 120 °C for 1 h. Ar was then flowed into the flask. OA and OAm (5 mL each) were injected at 120 °C. The PbI_2_ completely dissolved, leading to the formation of a clear solution. The Cs-oleate solution (~ 0.046 M, 5.9 mL), prepared as described above, was injected and the reaction mixture was quenched by immersion of the flask into an ice bath after a 5-min reaction. The crude nanorod solution was added into 200 mL methyl acetate and centrifuged at 8000 rpm for 5 min. The precipitates were dispersed in 6 mL hexane and 20 mL methyl acetate and then centrifuged at 8000 rpm for 2 min. The nanorods were dispersed in 10 mL of hexane and centrifuged again at 4000 rpm for 5 min. Finally, the as-prepared nanorods were dispersed in octane for further use.

### Device Fabrication

Heavily n-doped Si wafers with a thermally grown 300-nm-thick SiO_2_ layer were used as substrates. Before device fabrication, the substrates were cleaned using ultra-sonication consecutively with acetone, isopropanol, and deionized water for 30 min and finally dried by N_2_. The pre-cleaned substrates were immersed into a CsPbI_3_ nanorod solution (~ 20 mg mL^−1^ in octane) and pulled out at an optimized speed of 5 μm s^−1^ (SYDC-100H DIP COATER). The samples were then placed under vacuum for 2 h. A 30-nm-thick C8BTBT film was then deposited by vacuum evaporation, and 50-nm-thick Au source/drain electrodes on top of the hybrid films were thermally evaporated by shadow masks with a channel length of 5 μm and channel width of 1 mm. For the pure-CsPbI_3_-nanorod-based photodetectors, the nanorod solution was drop-cast directly on Si/SiO_2_ substrates with pre-patterned gold electrodes, followed by drying under vacuum for 1 h.

### Characterizations and Measurements

The surface morphologies of the CsPbI_3_ nanorod films were investigated by an atomic force microscope (AFM, SEIKO SPA-300HV) operated in the tapping mode. Ultraviolet–visible (UV–Vis) absorption spectra of thin films on quartz substrates were acquired using a Cary 60 UV–Vis spectrometer (Agilent Technologies). Powder X-ray diffraction (XRD) was performed using a DX-2700 X-ray diffractometer (Dandong Haoyunan Instrument Co. Ltd) with Cu K_α_ radiation (1.54 Å) at 40 kV and 50 mA. Transmission electron microscopy (TEM) and high-resolution TEM (HRTEM) images were recorded with a JEOL JEM-2100F TEM operated at 200 kV. The performances of the photodetectors were studied using a Keithley 4200 SCS. Monochromatic lights with different wavelengths were provided by a 300-W xenon lamp filtered with a double-grating monochromator (Omno 330150, Beijing NBeT, China). All of the measurements were taken in air at room temperature.

## Results and Discussion

**Fig. 1:**
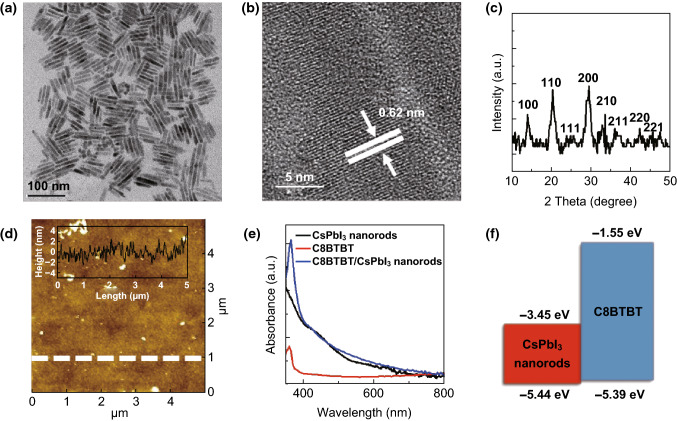
**a** TEM image, **b** HRTEM image, and **c** XRD patterns of CsPbI_3_ nanorods. **d** AFM image of the CsPbI_3_ nanorod film. The inset image presents a height profile of the film surface. **e** UV–Vis absorption spectra of CsPbI_3_ nanorods, C8BTBT, and C8BTBT/CsPbI_3_ nanorod films. **f** Energy level diagrams of C8BTBT and CsPbI_3_ nanorods

Figure 1a shows a TEM image of the as-synthesized CsPbI_3_ nanorods, indicating that the perovskites have a highly pure morphology with uniformly distributed diameters and lengths, with average values of ~ 10 and ~ 100 nm, respectively. The HRTEM image of the nanorods (Fig. 1b) shows an interplanar distance of 0.62 nm, which is consistent with the (100) plane of the cubic-phase CsPbI_3_ perovskite [36]. As shown in Fig. 1c, the structure of the CsPbI_3_ nanorods was studied using XRD, further confirming the cubic phase [37]. In this study, CsPbI_3_ nanorod thin films were fabricated by a one-step dip-coating method. An AFM analysis showed that the surface roughness *R*_a_ was approximately 0.87 nm (Fig. 1d). The film was sufficiently thin for a well alignment of the OSC molecules deposited on it, which provides the decent transistor performance of the hybrid device [38, 39]. A layer of C8BTBT was then evaporated on the CsPbI_3_ nanorod layer. The surface roughness of the CsPbI_3_ nanorod layer was essential for a high device performance.

An additional benefit of the use of C8BTBT as the OSC is that C8BTBT does not strongly adsorb visible light and hence has no responsivity to visible light, while the CsPbI_3_ nanorods have a broad absorption in both UV and visible regions. When the device was illuminated by visible light, only the CsPbI_3_ nanorods could act as the light adsorption materials, while C8BTBT acted only as a charge-transport material. It is beneficial to understand the underlying sensing mechanism of our devices. Figure 1e shows the UV–Vis absorbance spectra of a CsPbI_3_ nanorod film, C8BTBT film, and C8BTBT/CsPbI_3_ nanorod hybrid film. Although there was an intense absorption peak observed around 365 nm, the pure C8BTBT film did not exhibit an obvious absorption in the range of 400–800 nm. In contrast, the CsPbI_3_ nanorod film exhibited a strong and broad absorption in both UV and visible regions. The spectrum of the hybrid film included the absorptions of the C8BTBT and CsPbI_3_ nanorod films; the superimposed effect of the hybrid film obviously enhanced its light-absorption range.

A schematic band diagram of the CsPbI_3_ nanorods and C8BTBT is shown in Fig. 1f. The values of the conduction band (CB) and valence band (VB) of perovskite are at − 3.45 and − 5.44 eV, respectively [40]. The highest occupied molecular orbital (HOMO) and lowest unoccupied molecular orbital (LUMO) energy levels of C8BTBT are − 5.39 and − 1.55 eV, respectively [41]. Therefore, the CsPbI_3_ perovskite and C8BTBT form a type-II heterojunction [42]. The VB of CsPbI_3_ aligns well with the HOMO of C8BTBT, facilitating the injection of photogenerated holes from the perovskite into the OSC. Furthermore, the high LUMO energy level of C8BTBT effectively blocks electrons and prevents them diffusing into the OSC layer, thus efficiently reducing the photogenerated charge recombination.

**Fig. 2:**
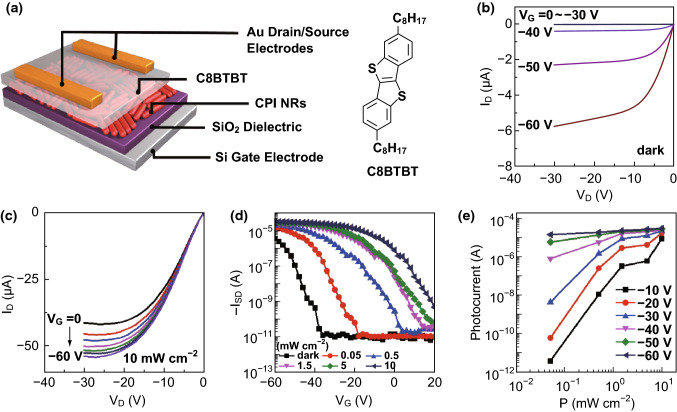
**a** Schematic diagram of the C8BTBT/CsPbI_3_ nanorod-based phototransistor. *I*_*D*_–*V*_*D*_ transistor characteristics of the hybrid phototransistor under a fixed illumination power intensity: **b** in the dark state and **c** under a white-light illumination of 10 mW cm^−2^. **d** Transfer characteristics (*V*_D_ = −30 V) under different illumination power densities. **e** Photocurrent as a function of the illumination power density for different gate voltages

The schematic diagram of the C8BTBT/CsPbI_3_ nanorod-based phototransistor is illustrated in Fig. 2a. Heavily n-doped silicon wafers coated with a 300-nm-thick SiO_2_ surface layer (capacitance: 10 nF cm^−2^) were used as the substrates and gate electrodes. The CsPbI_3_ perovskites were employed as the light absorber, while the OSC was used to facilitate the separation and transport of photogenerated electron–hole pairs. Au source/drain electrodes (50 nm) were thermally evaporated through shadow masks with a channel length of 5 μm and channel width of 1 mm. A power-tunable white LED with a wavelength range of 400–800 nm was used as the illumination source. As the photon energy (1.55–3.10 eV) was smaller than the bandgap of C8BTBT (3.84 eV), the OSC layer exhibited a limited photosensitive performance to the white light (Fig. S1). As shown in Fig. S2, the spectral response of the external quantum efficiency (EQE = *Rhc*/λe), where *hc*/λ is the photon energy, closely follows the absorption spectrum of the C8BTBT/CsPbI_3_ nanorod hybrid film. The output curves of the hybrid phototransistors were obtained in a dark state and under illumination of 10 mW cm^−2^, as shown in Fig. 2b, c, respectively. The devices exhibited p-type transistor *I*_*D*_–*V*_*D*_ characteristics in the dark state and under illumination, displaying both linear and saturation regions. The cut-off *V*_G_ of the device was − 40 V in the dark state. At the same applied voltage, the illumination increased the drain current and modulated the cut-off voltage of the device.

Figure 2d shows the transfer curves (*I*_*D*_–*V*_*G*_) of the hybrid phototransistors with a gate voltage sweeping from − 60 to 20 V and fixed drain voltage of − 30 V under illuminations of 0.05, 0.5, 1.5, 5, and 10 mW cm^−2^ and in the dark state, suggesting a typical p-type semiconductor behavior. The photocurrent (*I*_photo_=* I*_light_ − *I*_dark_) significantly enhanced with the increase in the illumination power density (Fig. 2e). The results show that a larger applied gate voltage leads to a larger increase in the photocurrent for the p-type phototransistors. Therefore, the photosensitive performances of the devices can be modulated by applying different gate voltages. Devices based on pure CsPbI_3_ nanorods were also fabricated by drop casting directly on Si/SiO_2_ substrates with pre-patterned gold electrodes; the *I*–*V* curves of the devices in the dark state and under an illumination of 5 mW cm^−2^ are shown in Fig. S3. The hybrid phototransistors exhibited not only a higher photocurrent, but also a significantly larger photocurrent to dark current ratio than those of the pure CsPbI_3_ nanorod devices, although the pure CsPbI_3_ nanorod devices exhibited a relatively high response speed [43].

**Fig. 3:**
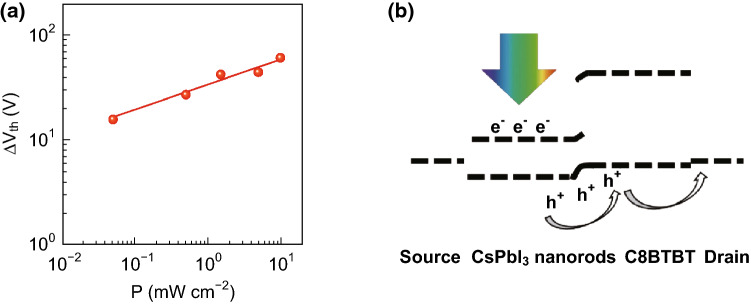
**a** Shift of the threshold voltage of the hybrid phototransistor as a function of the illumination power density. **b** Schematic of the photo-generated carrier transport in the hybrid phototransistor under illumination

With the increase in the light power density, the threshold voltage positively shifted. The Δ*V*_th_ is plotted as a function of the incident illumination power density in Fig. 3a. It can be fitted with Δ*V*_*th*_ = *αP*^*β*^, yielding *β* ≈ 0.24, where *β* is the power factor. The well-fitted line indicates that the shift of the threshold voltage was attributed to the photogating effect [44]. When the illumination power density increased, the Δ*V*_th_ tended to saturate, indicating that the photogenerated carriers in the light-absorber layer almost reached their maximum density. The *μ* of the device can be calculated from the saturation region of the transfer curve (Eq. 1) [45],

1 $$\mu = \frac{2L}{{WC_{i} }}\left( {\frac{{\partial \sqrt {I_{D} } }}{{\partial V_{G} }}} \right)^{2}$$

Figure S4 shows the illumination power density dependence of the normalized mobility. The *μ* slightly changed with the increase in the illumination power density; the field-effect mobility of the hybrid phototransistors can be regarded as a constant. Therefore, the enhancement in the photocurrent is attributed to the positively shifted threshold voltage rather than to change in the field-effect mobility. As shown in Fig. 3b, electron–hole pairs are generated only in the CsPbI_3_ nanorod layer when white light is incident on the device and dissociated into electrons and holes near the junction between the CsPbI_3_ nanorod and C8BTBT layers. The photogenerated holes in the perovskite layer can easily transfer to the C8BTBT layer, while the photoexcited electrons accumulated in the perovskite layer. When the hybrid devices are in the on-state, the photovoltaic effect is dominant as photo-voltage is induced by the large number of accumulated electrons under the source, and the illumination leads to a photoinduced positive shift of the threshold voltage, leading to a significant increase in the photocurrent. When the hybrid devices are in the off-state, the channel current exhibits a relatively small increase with the optical power owing to a photoconductive effect (Fig. S5) [46, 47].

**Fig. 4:**
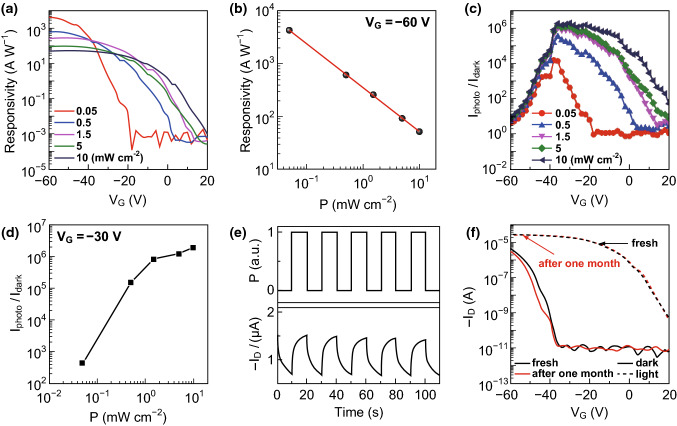
**a** Responsivity of the hybrid phototransistor as a function of the gate voltage for different illumination power densities. **b** Illumination power density dependence of the responsivity at *V*_*G*_ = −60 V, *V*_*D*_ = −30 V. **c** *I*_photo_/*I*_dark_ ratio as a function of the gate voltage for different illumination power densities. **d** Illumination-power-density dependence of the *I*_photo_/*I*_dark_ ratio at *V*_*G*_ = −60 V, *V*_*D*_ = −30 V. **e** Photoswitching characteristics of the hybrid phototransistor under an alternating dark and light illumination (0.5 mW cm^−2^) at *V*_*G*_ = −30 V, *V*_*D*_ = −30 V. **f** Transfer curves of the fresh hybrid phototransistor and device stored at an atmospheric environment (average temperature of 0 °C and average relative humidity of 50%) for 1 month

One of the important criteria of efficacy for phototransistors is the responsivity *R*, which can be expressed as Eq. 2:

2 $$R = \frac{{I_{\text{photo}} }}{PS}$$

where *P* is the incident illumination power density on the channel of the device, and *S* is the working area of the device. The values of *R* reveal to what extent the optical power is converted into photocurrent. The gate voltage dependences of the *R* for the devices illuminated with various light intensities are plotted in Fig. 4a, indicating that the *R* increased with the negative shift of the gate voltage when the devices were in the on-state. Figure 4b shows the illumination power density dependence of the *R*; the *R* decreased linearly with the incident power density (*r*^2^ = 0.99). An *R* as high as 4300 A W^−1^ can be obtained at *V*_*G*_ = −60 V, *V*_*D*_ = −30 V, as shown in Fig. 4b, which is comparable to the performances of the best perovskite-based photodetectors [22].

Another important parameter to evaluate the performance of the phototransistor is the photocurrent to dark current ratio (*I*_photo_/*I*_dark_). Figure 4c shows the *I*_photo_/*I*_dark_ ratio as a function of the gate voltage of the hybrid devices under various illumination intensities. When the devices were in the off-state under a sub-threshold voltage, the *I*_photo_*/I*_dark_ ratio increased with the increase in gate voltage, owing to the increased photocurrent with a contribution from the positively shifted threshold voltage. When the phototransistors were turned on by applying more negative gate voltages, a significant decrease in the *I*_photo_/*I*_dark_ ratio could be observed; the ratio disparities of the devices, caused by the different illumination intensities, rapidly diminished, as the electrical contribution to the charge carrier generation was dominant after the threshold was reached [48, 49]. The maximum *I*_photo_/*I*_dark_ ratio significantly increased with the incident illumination power increased; it could reach a value as high as 2.2 × 10^6^ under a gate voltage around − 30 V when the power density of the white light was 10 mW cm^−2^, as shown in Fig. 4d, which is among the highest values of previously reported perovskite-based photodetectors. The temporal photoresponses of the C8BTBT/CsPbI_3_ nanorod phototransistors were measured under an illumination of 0.5 mW cm^−2^ at *V*_*G*_ = −30 V, *V*_*D*_ = −30 V. Figure 4e shows the pulsed laser illumination (top) and temporal response of the photocurrent (bottom). The hybrid devices exhibited a good on–off switching. As shown in Fig. S6, an abrupt increase in the drain current can be observed when the hybrid devices were exposed to white light; the *I*_photo_/*I*_dark_ ratio was close to that in Fig. 4e. Owing to the high-level electron trapping capability in the CsPbI_3_ nanorod layer and at the C8BTBT/CsPbI_3_ nanorod interface, the trapped electrons remain in the thin film for a very long time with a low recombination rate and it is hard to reset the devices in a short time after removal of the incident light, keeping the devices in a metastable state with a high conductivity, leading to a decrease in the large drain current with a long relaxation time. The time responses of the photocurrent increase and decay were dominated by two components, which can be fitted with a double-exponential function (Eqs. 3 and 4) [44]:

3 $$\Delta I_{D} = \Delta I_{1} \exp (1 - t/\tau_{1} ) + \Delta I_{2} \exp (1 - t/\tau_{2} )$$

4 $$\Delta I_{D} = \Delta I_{3} ( - \exp ( - t/\tau_{3} )) + \Delta I_{4} ( - \exp ( - t/\tau_{4} ))$$

where Δ*I* is the change in the channel current and *τ* is the time constant (Fig. S7). The small relaxation times *τ*_1_ (0.63) and *τ*_3_ (0.54) correspond to the lifetime of holes in the CsPbI_3_ nanorod layer before they transferred to the C8BTBT layer, whereas the large relaxation times *τ*_2_ (4.24) and *τ*_4_ (9.62) represent the charge transfer between CsPbI_3_ nanorods [50].

The long-term stabilities of the hybrid phototransistors were also studied. As shown in Fig. 4f, the transfer curves of the hybrid phototransistors after they were stored in an atmospheric environment (average temperature of 20 °C and average relative humidity of 50%) for 1 month were almost perfectly overlapped with those of the fresh samples, indicating their excellent stabilities with respect to oxygen and moisture. Furthermore, Table S1 presents key parameters of the hybrid devices, compared with those of recently reported low-dimensional all-inorganic perovskite-based photodetectors; the photosensitivity, responsivity, and stability values of our devices are among the highest reported values. The excellent stabilities of the devices can be mainly attributed to the following factors. First, C8BTBT is an excellent OSC material possessing a good air stability. Second, 1D all-inorganic perovskites with a better crystallization tend to have higher stabilities than those of the organic–inorganic hybrid counterparts. Third, the OSC layer fabricated in the upper part of the hybrid layers protects the perovskite layer from atmospheric moisture.

## Conclusion

We demonstrated high-performance phototransistors based on the C8BTBT/CsPbI_3_ nanorod hybrid layers with high photosensitive performances and long-term stabilities in ambient conditions. The high-quality perovskite films were necessary to provide the desirable device performance. The perovskite layer could efficiently absorb the input illumination and generate a large amount of charge carriers, while a transport channel for the injection of photogenerated carriers and gate-tuning property was provided by the OSC layer. Owing to the photogating effect, the gate-tunable hybrid devices illuminated by white light exhibited outstanding photosensitivity performances with an *R* as high as 4300 A W^−1^ and ultra-high *I*_photo_/*I*_dark_ ratio of 2.2 × 10^6^, significantly higher than those of most of the previously reported perovskite-based photodetectors. Furthermore, the hybrid phototransistors possessed long-term stabilities owing to the high stabilities of the two materials and device structure under ambient conditions. Therefore, this study provides a strategy to combine the advantages of perovskite semiconductors and OSCs to obtain high-performance photodetectors.

## Electronic supplementary material

Below is the link to the electronic supplementary material.

Supplementary material 1 (PDF 278 kb)

## References

[1] Manser JS, Christians JA, Kamat PV (2016). Intriguing optoelectronic properties of metal halide perovskites. Chem. Rev..

[2] Saparov B, Mitzi DB (2016). Organic-inorganic perovskites: structural versatility for functional materials design. Chem. Rev..

[3] Kojima A, Teshima K, Shirai Y, Miyasaka T (2009). Organometal halide perovskites as visible-light sensitizers for photovoltaic cells. J. Am. Chem. Soc..

[4] McMeekin DP, Sadoughi G, Rehman W, Eperon GE, Saliba M (2016). A mixed-cation lead mixed-halide perovskite absorber for tandem solar cells. Science.

[5] Deng W, Xu XZ, Zhang XJ, Zhang YD, Jin XC, Wang L, Lee ST, Jie JS (2016). Organometal halide perovskite quantum dot light-emitting diodes. Adv. Funct. Mater..

[6] Cho H, Kim YH, Wolf C, Lee HD, Lee TW (2018). Improving the stability of metal halide perovskite materials and light-emitting diodes. Adv. Mater..

[7] Fu Y, Zhu H, Schrader AW, Liang D, Ding Q, Joshi P, Hwang L, Zhu XY, Jin S (2016). Nanowire lasers of formamidinium lead halide perovskites and their stabilized alloys with improved stability. Nano Lett..

[8] Wang H, Kim DH (2017). Perovskite-based photodetectors: materials and devices. Chem. Soc. Rev..

[9] Chen S, Shi G (2017). Two-dimensional materials for halide perovskite-based optoelectronic devices. Adv. Mater..

[10] Chen C, Zhang X, Wu G, Li H, Chen H (2016). Visible-light ultrasensitive solution-prepared layered organic-inorganic hybrid perovskite field-effect transistor. Adv. Opt. Mater..

[11] Tiep NH, Ku Z, Fan HJ (2016). Recent advances in improving the stability of perovskite solar cells. Adv. Energy Mater..

[12] Yi C, Luo J, Meloni S, Boziki A, Ashari-Astani N, Gratzel C, Zakeeruddin SM, Rothlisberger U, Gratzel M (2016). Entropic stabilization of mixed A-cation ABX_3_ metal halide perovskites for high performance perovskite solar cells. Energy Environ. Sci..

[13] Eaton SW, Lai M, Gibson NA, Wong AB, Dou L, Ma J, Wang L-W, Leone SR, Yang P (2016). Lasing in robust cesium lead halide perovskite nanowires. Proc. Natl. Acad. Sci. USA.

[14] Lai M, Kong Q, Bischak CG, Yu Y, Dou L, Eaton SW, Ginsberg NS, Yang P (2017). Structural, optical, and electrical properties of phase-controlled cesium lead iodide nanowires. Nano Res..

[15] Lu C, Li H, Kolodziejski K, Dun C, Huang W, Carroll D, Geyer SM (2018). Enhanced stabilization of inorganic cesium lead triiodide (CsPbI_3_) perovskite quantum dots with tri-octylphosphine. Nano Res..

[16] Zhang XS, Wang Q, Jin ZW, Zhang JR, Liu SF (2017). Stable ultra-fast broad-bandwidth photodetectors based on α-CsPbI_3_ perovskite and NaYF4:Yb, Er quantum dots. Nanoscale.

[17] Waleed A, Tavakoli MM, Gu L, Hussain S, Zhang D, Poddar S, Wang Z, Zhang R, Fan Z (2017). All inorganic cesium lead iodide perovskite nanowires with stabilized cubic phase at room temperature and nanowire array-based photodetectors. Nano Lett..

[18] Chen H, Liu H, Zhang Z, Hu K, Fang X (2016). Nanostructured photodetectors: from ultraviolet to terahertz. Adv. Mater..

[19] Li M, Zhang X, Du Y, Yang P (2017). Colloidal CsPbX3 (X = Br, I, Cl) NCs: morphology controlling, composition evolution, and photoluminescence shift. J. Lumin..

[20] Zhou H, Yuan S, Wang X, Xu T, Wang X (2017). Vapor growth and tunable lasing of band gap engineered cesium lead halide perovskite micro/nanorods with triangular cross section. ACS Nano.

[21] Fang F, Chen W, Li Y, Liu H, Mei M (2018). Employing polar solvent controlled ionization in precursors for synthesis of high-quality inorganic perovskite nanocrystals at room temperature. Adv. Funct. Mater..

[22] Yang T, Zheng Y, Du Z, Liu W, Yang Z (2018). Superior photodetectors based on all-inorganic perovskite CsPbI_3_ nanorods with ultrafast response and high stability. ACS Nano.

[23] Tang X, Zu Z, Shao H, Hu W, Zhou M (2016). All-inorganic perovskite CsPb(Br/I)_3_ nanorods for optoelectronic application. Nanoscale.

[24] Li XM, Cao F, Yu DJ, Chen J, Sun ZG (2017). All inorganic halide perovskites nanosystem: synthesis, structural features, optical properties and optoelectronic applications. Small.

[25] Chu YL, Wu XH, Lu JJ, Liu DP, Du J, Zhang GQ, Huang J (2016). Photosensitive and flexible organic field-effect transistors based on interface trapping effect and their application in 2D imaging array. Adv. Sci..

[26] Wu XH, Ma Y, Zhang GQ, Chu YL, Du J (2015). Thermally stable, biocompatible, and flexible organic field-effect transistors and their application in temperature sensing arrays for artificial skin. Adv. Funct. Mater..

[27] Huang J, Zhu HL, Chen YC, Preston C, Rohrbach K, Cumings J, Hu LB (2013). Highly transparent and flexible nanopaper transistors. ACS Nano.

[28] Wu X, Mao S, Chen J, Huang J (2018). Strategies for improving the performance of sensors based on organic field-effect transistors. Adv. Mater..

[29] Wang W, Wang L, Dai G, Deng W, Zhang X, Jie J, Zhang X (2017). Controlled growth of large-area aligned single-crystalline organic nanoribbon arrays for transistors and light-emitting diodes driving. Nano-Micro Lett..

[30] Yu P, Hu K, Chen H, Zheng L, Fang X (2017). Novel p–p heterojunctions self-powered broadband photodetectors with ultrafast speed and high responsivity. Adv. Funct. Mater..

[31] Gao H, Feng J, Zhang B, Xiao C, Wu Y (2017). Capillary-bridge mediated assembly of conjugated polymer arrays toward organic photodetectors. Adv. Funct. Mater..

[32] Tong S, Sun J, Wang C, Huang Y, Zhang C (2017). High-performance broadband perovskite photodetectors based on CH_3_NH_3_PbI_3_/C8BTBT heterojunction. Adv. Electron. Mater..

[33] Huang J, Du J, Cevher Z, Ren YH, Wu XH, Chu YL (2017). Printable and flexible phototransistors based on blend of organic semiconductor and biopolymer. Adv. Funct. Mater..

[34] Wu G, Chen C, Liu S, Fan C, Li H, Chen H (2015). Solution-grown organic single-crystal field-effect transistors with ultrahigh response to visible-blind and deep UV signals. Adv. Electron. Mater..

[35] Swarnkar A, Marshall AR, Sanehira EM, Chernomordik BD, Moore DT, Christians JA, Chakrabarti T, Luther JM (2016). Quantum dot-induced phase stabilization of α-CsPbI_3_ perovskite for high-efficiency photovoltaics. Science.

[36] Zhang X, Zhang J, Phuyal D, Du J, Tian L (2017). Inorganic CsPbI_3_ perovskite coating on pbs quantum dot for highly efficient and stable infrared light converting solar cells. Adv. Energy Mater..

[37] Protesescu L, Yakunin S, Bodnarchuk MI, Krieg F, Caputo R, Hendon CH, Yang RX, Walsh A, Kovalenko MV (2015). Nanocrystals of cesium lead halide perovskites (CsPbX_3_, X = Cl, Br, and I): novel optoelectronic materials showing bright emission with wide color gamut. Nano Lett..

[38] Wang CL, Dong HL, Hu WP, Liu YQ, Zhu DB (2012). Semiconducting π-conjugated systems in field-effect transistors: a material odyssey of organic electronics. Chem. Rev..

[39] Chen Y, Chu Y, Wu X, Ou-Yang W, Huang J (2017). High-performance inorganic perovskite quantum dot-organic semiconductor hybrid phototransistors. Adv. Mater..

[40] De A, Mondal N, Samanta A (2018). Hole transfer dynamics from photoexcited cesium lead halide perovskite nanocrystals: 1-aminopyrene as hole acceptor. J. Phys. Chem. C (Article ASAP).

[41] He D, Zhang Y, Wu Q, Xu R, Nan H (2014). Two-dimensional quasi-freestanding molecular crystals for high-performance organic field-effect transistors. Nat. Commun..

[42] Li F, Wang H, Kufer D, Liang L, Yu W (2017). Ultrahigh carrier mobility achieved in photoresponsive hybrid perovskite films via coupling with single-walled carbon nanotubes. Adv. Mater..

[43] Lv LF, Xu YB, Fang HH, Luo WJ, Xu FJ (2016). Generalized colloidal synthesis of high-quality, two-dimensional cesium lead halide perovskite nanosheets and their applications in photodetectors. Nanoscale.

[44] Sun Z, Li J, Yan F (2012). Highly sensitive organic near-infrared phototransistors based on poly(3-hexylthiophene) and PbS quantum dots. J. Mater. Chem..

[45] Horowitz Gilles (1998). Organic Field-Effect Transistors. Advanced Materials.

[46] Xu Y, Berger PR, Wilson JN, Bunz UHF (2004). Photoresponsivity of polymer thin-film transistors based on polyphenyleneethynylene derivative with improved hole injection. Appl. Phys. Lett..

[47] Noh YY, Kim DY, Yoshida Y, Yase K, Jung BJ, Lim E, Shim HK (2005). High-photosensitivity p-channel organic phototransistors based on a biphenyl end-capped fused bithiophene oligomer. Appl. Phys. Lett..

[48] Ljubic D, Smithson CS, Wu Y, Zhu S (2016). Effect of polymer binders on uv-responsive organic thin-film phototransistors with benzothienobenzothiophene semiconductor. ACS Appl. Mater. Interfaces.

[49] Baeg KJ, Binda M, Natali D, Caironi M, Noh YY (2013). Organic light detectors: photodiodes and phototransistors. Adv. Mater..

[50] Tetsuka H, Nagoya A, Fukusumi T, Matsui T (2016). Molecularly designed, nitrogen-functionalized graphene quantum dots for optoelectronic devices. Adv. Mater..

