# Supplementary material for: Hybrid Field-Effect Transistors and Photodetectors Based on Organic Semiconductor and CsPbI3 Perovskite Nanorods Bilayer Structure
Source: Nanomicro Lett. 2018 Jun 23;10(4):57. doi: 10.1007/s40820-018-0210-8 (PMC6199102; doi:10.1007/s40820-018-0210-8)
Supplement: Supplementary file 1 — Supplementary material 1 (PDF 278 kb) [file 40820_2018_210_MOESM1_ESM.pdf]

Supporting Information for

Hybrid Field-Effect Transistors and Photodetectors Based on Organic Semiconductor and $CsPbI_3$ Perovskite Nanorods Bilayer Structure

Yantao Chen[2], Xiaohan Wu[2], Yingli Chu[2], Jiachen Zhou[2], Bilei Zhou[2], Jia Huang[1, 2, *]

[1]Key Laboratory of Road and Traffic Engineering of Ministry of Education, Tongji University, Shanghai 201804, People's Republic of China

[2]Interdisciplinary Materials Research Center, School of Materials Science and Engineering, Tongji University, Shanghai 201804, People's Republic of China

*Corresponding author. E-mail: huangjia@tongji.edu.cn (Jia Huang)

Supplementary Figures

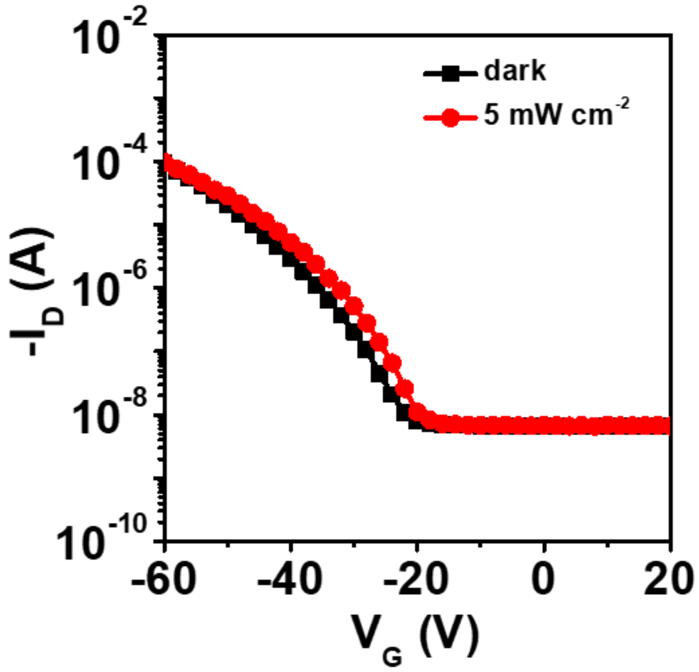

Fig. S1 Photoresponse properties for transistors based on C8BTBT in the dark and under light illumination (10 mW cm^{-2}) at V_D = -30 V

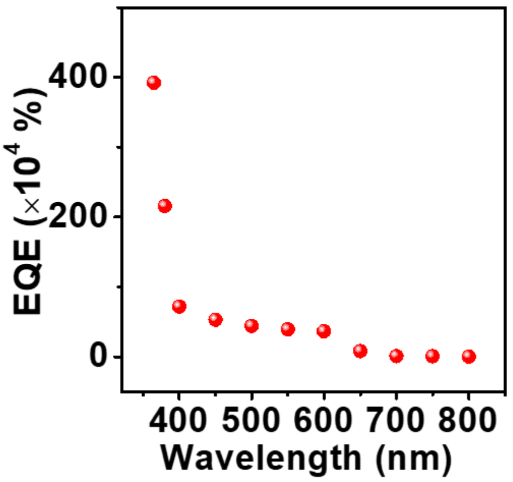

Fig. S2 Calculated *EQE* of the hybrid phototransistors as a function of the illumination wavelength under 0.5 mW cm^{-2} at V_G = -60 V, V_D = -30 V

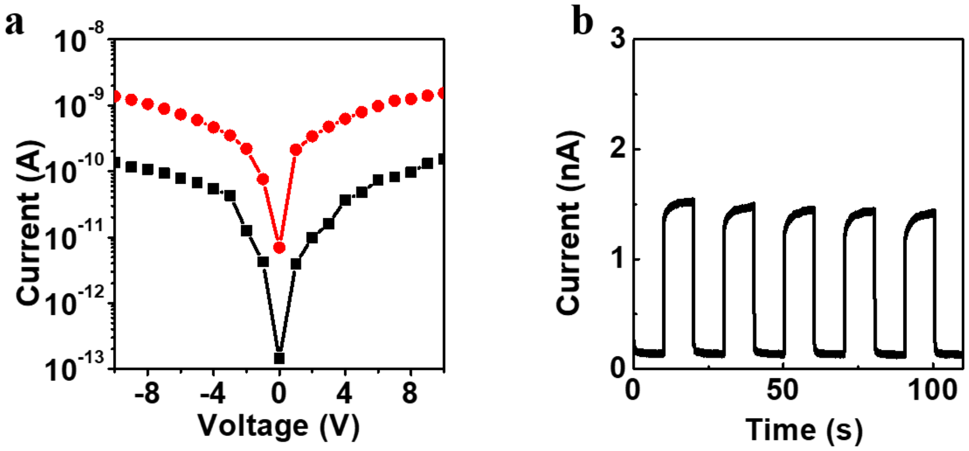

Fig. S3 **a** *I-V* curves of the only $CsPbI_3$ nanorods-based photodetectors measured in dark and under 5 mW cm^{-2}. **b** Time-dependent photoresponse of the only $CsPbI_3$ nanorods-based photodetectors measured in dark and under 5 mW cm^{-2} at 10 V

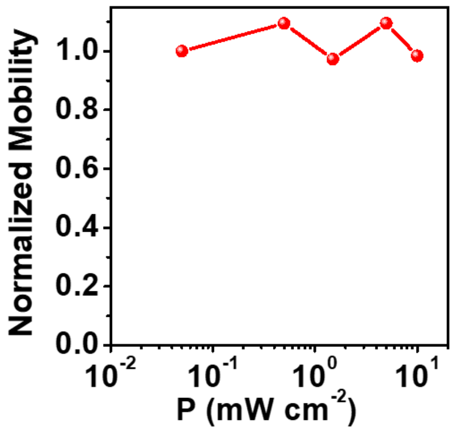

Fig. S4 Normalized mobility of the hybrid phototransistors versus the power intensity at V_D = -30 V

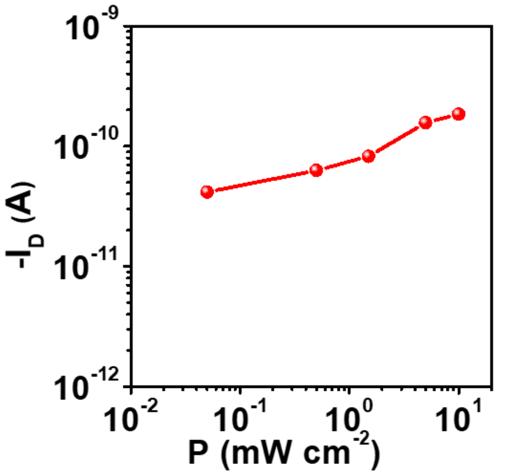

Fig. S5 The output current in off-state of the hybrid phototransistor as a function of the power intensity at V_D = -30 V

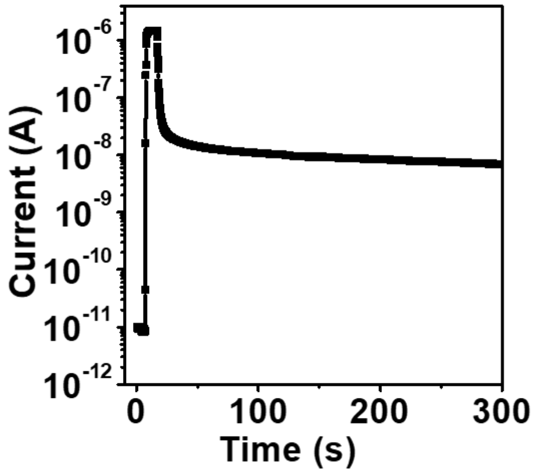

Fig. S6 10 s white light irradiation of the hybrid devices under 0.5 mW cm^{-2} followed by removal of light incident to allow relaxation for 5 min

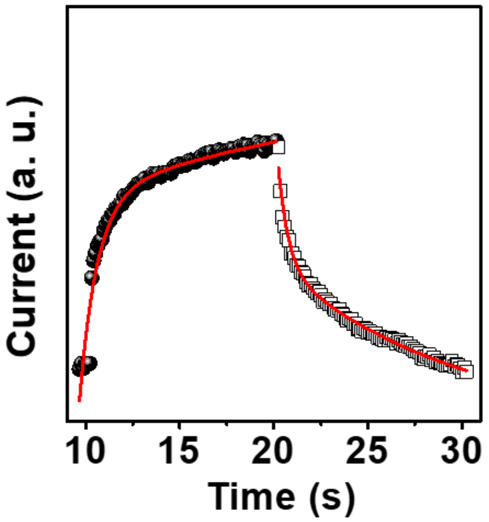

Fig. S7 The photoresponse curves of the hybrid phototransistor and the fitted lines

Table S1 Performance comparison of our C8BTBT/$CsPbI_3$ nanorods-based photodetectors with low-dimensional all inorganic perovskite-based photodetectors in literatures

Material	Light source	R($A\ W^{-1}$)	I_{photo}/I_{dark}	Stability	Ref.
$CsPbI_3$ nanorods	405 nm	2.92×10^3	3×10^3	1 week	[1]
$CsPbI_3$ nanoarrays	N/A	0.0067	N/A	N/A	[2]
$CsPbI_3$ nanowires	White light	N/A	100	4 weeks	[3]
$CsPbI_3$ nanocrystals films	405 nm	N/A	10^5	N/A	[4]
$CsPbI_3$ QDs /$NaYF_4$:Yb,Er QDs	525 nm	1.5	10^4	60 days	[5]
$CsPb(Br/I)_3$ nanorods	532 nm	N/A	10^3	N/A	[6]
**$CsPbI_3$ nanorods /C8BTBT**	**White light**	**5.3×10^3**	**2.2×10^6**	**1 month**	**This work**

Supplementary References

[1] T. Yang, Y. Zheng, Z. Du, W. Liu, Z. Yang et al., Superior photodetectors based on all-inorganic perovskite $CsPbI_3$ nanorods with ultrafast response and high stability. ACS Nano. **12**(2), 1611-1617 (2018). https://doi.org/10.1021/acsnano.7b08201

[2] A. Waleed, M.M. Tavakoli, L. Gu, S. Hussain, D. Zhang et al., All inorganic cesium lead iodide perovskite nanowires with stabilized cubic phase at room temperature and nanowire array-based photodetectors. Nano Lett. **17**(8), 4951-4957 (2017). https://doi.org/10.1021/acs.nanolett.7b02101

[3] Lai M, Kong Q, Bischak CG, Yu Y, Dou L, Eaton SW, Ginsberg NS, Yang P. Structural, optical, and electrical properties of phase-controlled cesium lead iodide nanowires. Nano Res. **10**(4), 1107-1114 (2017). https://doi.org/10.1007/s12274-016-1415-0

[4] P. Ramasamy, D.H. Lim, B. Kim, S.H. Lee, M.S. Lee, J.S. Lee, All-inorganic cesium lead halide perovskite nanocrystals for photodetector applications. Chem. Commun. **52**(10), 2067-2070 (2016). https://doi.org/ 10.1039/C5CC08643D

[5] X.S. Zhang, Q. Wang, Z.W. Jin, J.R. Zhang, S.F. Liu, Stable ultra-fast broad-bandwidth photodetectors based on α-$CsPbI_3$ perovskite and $NaYF_4$:Yb,Er quantum dots. Nanoscale **9**(19), 6278-6285 (2017). https://doi.org/10.1039/c7nr02010d

[6] X. Tang, Z. Zu, H. Shao, W. Hu, M. Zhou et al., All-inorganic perovskite $CsPb(Br/I)_3$ nanorods for optoelectronic application. Nanoscale **8**(33), 15158-15161 (2016). https://doi.org/10.1039/c6nr01828a
